# EXPERIENTIAL AVOIDANCE IN A SAMPLE OF DEMENTIA CAREGIVERS

**DOI:** 10.1093/geroni/igz038.1815

**Published:** 2019-11-08

**Authors:** Amy M Houston, Elisabeth Harfmann, Amy Olzmann, Glenna Brewster, Hannah Ottmar

**Affiliations:** 1 Milwaukee VA, Milwaukee, Wisconsin, United States; 2 Milwaukee VA, Milwaukee, Virginia, United States; 3 Baltimore VA, Baltimore VA, United States; 4 Emory University Nell Hodgson Woodruff School of Nursing, Atlanta, Georgia, United States; 5 Xavier University, Cincinnati, Ohio, United States

## Abstract

Given the rapidly changing demographics, there will be an increasing number of individuals with dementia who will need significant support from informal caregivers. Providing care for an individual with dementia has been associated with negative outcomes in a number of domains including physical health, mental health, financial status and social functioning. There is a small but growing base of literature suggesting that fostering psychological flexibility, including acceptance, with dementia caregivers may be a helpful intervention. Experiential avoidance, which is the less adaptive alternative to acceptance, is the aversion from negative internal experiences including thoughts, feeling and physical sensations. Experiential avoidance has been found to be significantly related to depression and negative affect. The present study utilized online dementia caregiver support group samples (n = 158) to evaluate the relationship between experiential avoidance and general demographics, other aspects of psychological flexibility, and caregiver distress. Experiential avoidance was positively correlated with cognitive fusion (r(134) = .231, p < .01), caregiver burden (r(127) = .258, p < .01), and distress associated with dementia related behaviors (r(140) = .225, p < .01). Experiential avoidance was negatively correlated with engaged living (r(133) = -.244, p < .01), mindfulness (r(123) = -.187, p < .05), and self-rated health (r(138) = -.193, p < .05). Additionally, experiential avoidance was significantly higher for male caregivers (t(136)=2.462, p=.015) and those age 65 and over (t(134)=-2.421, p=.017). These findings support previous research that suggests experiential avoidance may be an important construct to target in future interventions with dementia caregivers.

